# Spinal manipulation and exercise for low back pain in adolescents: study protocol for a randomized controlled trial

**DOI:** 10.1186/2045-709X-22-21

**Published:** 2014-05-23

**Authors:** Craig Schulz, Brent Leininger, Roni Evans, Darcy Vavrek, Dave Peterson, Mitchell Haas, Gert Bronfort

**Affiliations:** 1 Wolfe-Harris Center for Clinical Studies, Northwestern Health Sciences University, 2501 W 84th St, Bloomington, MN 55431, USA; 2 Center for Outcome Studies, University of Western States, 2900 NE 132nd Ave, Portland, OR 97230, USA; 3 Division of Chiropractic Sciences, University of Western States, 2900 NE 132nd Ave, Portland, OR 97230, USA

**Keywords:** Back pain, Pain, Spinal manipulation, Musculoskeletal manipulations, Exercise, Randomized controlled trial, Adolescent

## Abstract

**Background:**

Low back pain is among the most common and costly chronic health care conditions. Recent research has highlighted the common occurrence of non-specific low back pain in adolescents, with prevalence estimates similar to adults. While multiple clinical trials have examined the effectiveness of commonly used therapies for the management of low back pain in adults, few trials have addressed the condition in adolescents. The purpose of this paper is to describe the methodology of a randomized clinical trial examining the effectiveness of exercise with and without spinal manipulative therapy for chronic or recurrent low back pain in adolescents.

**Methods/design:**

This study is a randomized controlled trial comparing twelve weeks of exercise therapy combined with spinal manipulation to exercise therapy alone. Beginning in March 2010, a total of 184 participants, ages 12 to 18, with chronic or recurrent low back pain are enrolled across two sites. The primary outcome is self-reported low back pain intensity. Other outcomes include disability, quality of life, improvement, satisfaction, activity level, low back strength, endurance, and motion. Qualitative interviews are conducted to evaluate participants’ perceptions of treatment.

**Discussion:**

This is the first randomized clinical trial assessing the effectiveness of combining spinal manipulative therapy with exercise for adolescents with low back pain. The results of this study will provide important evidence on the role of these conservative treatments for the management of low back pain in adolescents.

**Trial registration:**

(ClinicalTrials.gov NCT01096628).

## Background

### Low back pain prevalence and burden in adolescents

Low back pain (LBP) in adolescents has become increasingly recognized as a public health concern, with an estimated one year prevalence of 34% [1]. Often beginning at a young age, the prevalence of LBP quickly mirrors that of adults by the late teenage years [2]. The natural history of LBP in adolescents is also similar to adults with recurrent symptoms occurring in over one half of patients [3]. Importantly, recurrent LBP in adolescence is highly associated with continued pain in adulthood [4,5].

While the prevalence of LBP steadily increases with age during childhood, the number of adolescents seeking care does not dramatically increase until mid-adolescence [6]. Care-seeking for LBP in childhood is strongly influenced by pain intensity and limitations in daily activities [7]. Approximately one-quarter of adolescents with LBP miss school because of their LBP condition [8].

Although little is currently known regarding the societal cost of LBP in adolescents, the disease burden in adulthood is substantial. The 2010 Global Burden of Disease study ranked LBP as the leading cause of years lived with disability in the world [9]. After respiratory disorders, LBP causes more short-term work absence that any other condition [10]. In the US alone, costs associated with LBP are estimated between $20 and $120 billion annually and are increasing [11,12]. In addition, there are growing concerns regarding the overutilization and safety of frequently prescribed medical treatments, particularly in light of the lack of evidence to support their effectiveness [13].

Recognizing that LBP begins at an early age and becomes burdensome and costly in adulthood, research focusing on conservative management strategies for adolescents with LBP is urgently needed.

### Treatment of adolescent LBP

Whereas multiple randomized trials have examined the effectiveness of commonly used conservative treatments for LBP in adults [14-17], few have been performed in adolescent LBP populations. Three small randomized clinical trials have evaluated exercise as a treatment approach for adolescent LBP [18-20]. Collectively, the published trials to date suggest exercise may be effective in reducing pain and disability in adolescents with LBP, but larger, high quality trials are needed. Interestingly, although spinal manipulative therapy (SMT) is commonly used for children with spine conditions [21], no randomized trials examining the effectiveness of SMT for adolescent LBP have been published to date [22].

### Study aims

Our primary aim is to determine the relative clinical effectiveness of 1) exercise with SMT versus 2) exercise alone in 184 adolescents with sub-acute recurrent (2-12 weeks) or chronic (>12 weeks) LBP, measured at 12, 26, and 52 weeks. The primary outcome measure is patient-rated LBP at these time points. Our hypothesis is that the addition of SMT to exercise will reduce pain more than exercise alone.

Our secondary aims are to assess between-group differences in disability, quality of life, improvement, satisfaction, activity level, dynamic lumbar motion, and trunk muscle endurance. In addition, we will assess the participant’s perceptions of treatment using qualitative interviews.

## Methods/design

### Design & setting

This trial began in March 2010, and is a two-site, parallel-group randomized controlled trial. Participants are being recruited at Northwestern Health Sciences University in Bloomington, MN and the University of Western States in Portland, OR. All treatments are provided within university-associated outpatient clinics.

### Ethical approval

Ethical approval has been granted by the Institutional Review Boards of the participating institutions (Project ID: 1-77-10-09).

### Recruitment

Potential subjects are recruited from the Minneapolis/St. Paul, MN and Portland, OR metropolitan areas using multiple methods. The primary recruitment method is targeted mailings to households with adolescents. Other recruitment methods include internet advertising through Facebook and Craigslist, newspaper advertising, flyers, and targeted referral letters to local physicians specializing in pediatrics or musculoskeletal conditions and coaches of sport programs at local schools.

### Participants

Adolescents, age 12 to18, with a history of sub-acute recurrent or chronic low back pain are eligible to participate. The inclusion/exclusion criteria are described in Table 1.

**Table 1:** Inclusion and exclusion criteria

**Inclusion criteria**	**Exclusion criteria**
• 12 to 18 years old	• Spinal manipulative therapy or exercise therapy in the previous month
• Either:	
◦ Sub-acute recurrent non-specific low back pain defined as current episode of 2 to 12 weeks duration AND an additional 2 week episode of back pain in the past year	• Ongoing treatment for low back pain by other health care providers
	• Other serious physical or mental health conditions (e.g., uncontrolled diabetes, cancer)
OR	• Contraindications to study treatments including acute disc herniation, clinical
◦ Chronic non-specific low back pain defined as a current episode ≥ 12 weeks duration	
	• instability of the lumbar spine, or inflammatory arthritides
• Low back pain intensity ≥ 3 on 0 to 10 numerical rating scale	• Benign joint hypermobility syndrome
• Stable prescription medication plan (no changes to prescription medications that affect musculoskeletal pain in the previous month)	

### Baseline assessments

**Figure 1:**
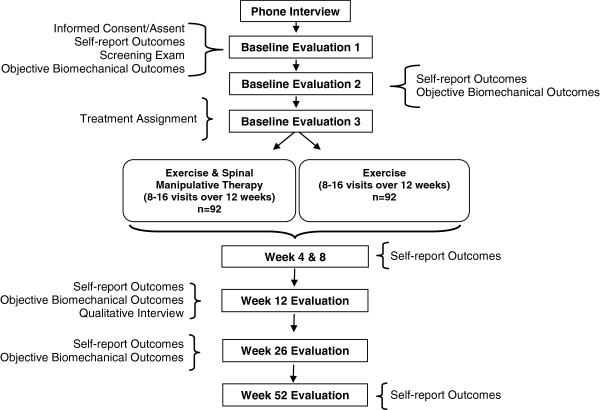
**Study flow diagram. Self-report Outcomes:** Primary and secondary outcomes (e.g., Pain, Disability). **Objective Biomechanical Outcomes:** Torso muscle strength & endurance; Continuous lumbar motion; Accelerometer readings for 7 consecutive days. **Qualitative Interview:** Face-to-face interviews assessing treatment experience and expectations.

Interested individuals are assessed for eligibility and compliance during a scripted telephone interview followed by three distinct baseline evaluations. For participants ages 12-17, written assent is obtained in addition to written informed consent from a legal guardian. Participants 18 years of age provide written informed consent. Individuals complete a clinical health history and physical exam. Plain film radiographs are taken if suspicion of a specific cause of LBP is present. To maintain consistency across sites, study clinicians and investigators, who are blinded to upcoming treatment assignment, meet (in-person and by teleconference) to review each case to determine eligibility. Self-report outcome measures and blinded objective assessments of lumbar spine motion, strength, and endurance are completed at the first two baseline evaluations. An accelerometer is issued to potential participants to monitor their physical activity level for the week prior to treatment assignment. Participants are assigned to study treatments at the third baseline evaluation. The participant’s flow through baseline evaluations and treatment is shown in Figure 1.

### Treatment allocation & concealment

As participants become eligible, study staff masked to upcoming treatment assignments use a dynamic allocation (rank-order minimization) computer program to assign subjects to treatment [23-25]. Gender, age, pain duration, and intensity are entered into the program to balance patient characteristics between groups.

A computer-generated random allocation sequence secured in sealed, opaque, sequentially numbered envelopes is used to randomize the first six participants at each site to provide a “seed” group of participants for the dynamic allocation program. Envelopes are also used as a back-up in the event the treatment allocation program becomes unavailable.

### Interventions

All interventions are provided by study staff at Northwestern Health Sciences University and the University of Western States research clinics. The physical nature of the treatments prevents blinding of participants and providers to treatment assignment. Participants are asked not to receive care for their LBP condition from other providers during the active treatment phase (12 weeks). Data regarding non-study care is collected in the self-report questionnaires.

Treatment providers have been trained in protocols to ensure standardized intervention and documentation. To facilitate protocol adherence, clinical records are monitored weekly by research staff. Non-adherent providers and patients are contacted using various methods (e.g., in-person, by phone, or email) to resolve compliance issues.

#### Exercise therapy (ET)

There are three components to the ET program: self-care education, supervised exercise visits, and home exercise. The overall objectives are to help adolescents manage their LBP and prevent future LBP recurrences. The total number of visits ranges from 8 to 16, depending on the patient’s needs (e.g., ability to perform exercises independently). Experienced chiropractors and exercise therapists have been trained and certified to deliver the program.

The details of the exercise program are outlined in Table 2. Each visit starts with 10 minutes of self-care education to help patients establish and monitor goals aligned with the exercise program and enhance their understanding of LBP. Participants are taught the importance of movement and activity, pain management techniques, and methods for developing spinal posture awareness during activities of daily living (e.g., lifting, pushing, pulling, sitting, getting out of bed, and using a backpack). Individuals are provided printed instructions and photos of exercises in addition to a modified *Back in Action*[26] binder, which emphasizes movement and restoration of normal function and fitness [27,28]. The supervised exercises begin with a 5-minute, light aerobic ◦warm up, followed by stretching and strengthening exercises (bridge, abdominal crunches, quadruped, side bridge, and back extensions). Participants begin with exercises appropriate for their fitness level and gradually progress in difficulty by changing body position and/or using a labile surface (i.e., gym ball). Participants are encouraged to perform the same exercises at home combined with 20-40 minutes of aerobic activity two times per week.

**Table 2:** Treatment interventions

**Intervention**	**Spinal Manipulative Therapy (SMT)**	**Exercise Therapy (ET)**
**Type**	• High velocity, low amplitude SMT preferred	**ET Visit**
		Self-care education
	• Other manual therapies if needed	• Supervised exercise
	◦ Low velocity, low amplitude SMT or mobilization	
		◦ Aerobic warm up
	◦ Flexion-distraction manipulation	
	◦ Drop-table assisted SMT	◦ Stretching (cat/camel, piriformis, hamstring, laying back rotation)
	• Up to 5 minutes adjunct therapies to facilitate SMT:	
	◦ Light soft tissue massage, active and passive stretching, ischemic compression of tender points, ice and heat	◦ Strengthening (bridge, abdominal curl, back extension, side bridge, quadruped, and squats) with changes in body positioning and addition of labile surface (i.e., gym ball) for progressions
		**Home Exercise**
		• Instructions provided at treatment visits and supplemented by take home materials
**Design & delivery format**	• Individualized: number of visits, spinal levels treated, SMT and manual therapy technique used and adjunct therapies determined by provider according to patient needs and tolerance	**ET Visit**
		• Supervised
		• Individualized: number of visits, exercise progressions determined by provider
		**Home Exercise**
		• Unsupervised
**Delivery method**	• One-on-one treatment visit	**ET Visit**
	• Treatment provided by licensed chiropractor	• One-on-one exercise therapy visit
		• Instructions and supervision provided by licensed chiropractor or exercise therapist
		**Home Exercise**
		• Instructions provided at treatment visits
		• Instructions supplemented by take home materials (exercise photos, modified *Back in Action*[26] book)
**Dose**	• 8 to 16 treatment visits	**ET Visit**
	• 10 to 20 minutes per visit	• 8 to 16 visits
	• Maximum frequency: 2 times/week	
		• 45 minutes per visit
		◦ 10 minutes self-care education
		◦ 5 minutes aerobic exercise warm up
		◦ 30 minutes supervised exercise
		▪ Stretching: 1 set; 3 reps
		▪ cat/camel; 1 rep each side for other stretches
		▪ Strengthening: 2 sets; 16 to 20 repetitions each exercise
		**Home Exercise**
		• Maximum frequency: 2 times/week

#### Exercise therapy combined with spinal manipulative therapy (ET + SMT)

Participants in the ET + SMT group participate in the same ET program described above. In addition, they take part in 8 to 16, 10- to 20-minute sessions of SMT over 12 weeks (Table 2). Previous research has shown that 9 to 12 treatments are more beneficial than 3 to 6 treatments for adults with LBP [29]. The number and frequency of treatments is determined by the individual chiropractor based on patient-rated symptoms and exam findings [30]. SMT is provided by experienced chiropractors following standardized treatment protocols. The preferred treatment consists of a high-velocity, low amplitude thrust SMT. The treating chiropractor has the option to perform other techniques (e.g., flexion-distraction, spinal mobilization, or neuromuscular techniques) [31], as indicated. Light soft tissue techniques (i.e., active and passive muscle stretching, hot and cold packs, and ischemic compression of tender points) may be used as needed to facilitate the manual therapy.

#### Modification and discontinuation of treatment

Prescription-strength nonsteroidal anti-inflammatory rescue medications are available for patients experiencing severe pain and are prescribed as needed by a study medical doctor. Any patient who demonstrates progressive neurological signs or severe intractable pain is removed from study treatment and referred for orthopedic consultation. Participants who receive medications or are discontinued from treatment will be included in the intention-to-treat analyses.

### Outcomes

Patient self-report outcomes are collected at the first two baseline visits and at 4, 8, 12, 26, and 52 weeks after randomization. The self-report outcomes are based on the Pediatric Initiative on Methods, Measurement, and Pain Assessment in Clinical Trials (PedIMMPACT) groups’ recommendations [32]. Objective spinal biomechanical outcome measures are collected at the first two baseline visits and at 12 and 26 weeks after study enrollment by examiners blinded to treatment assignment. The participant’s physical activity level is assessed at baseline and at weeks 12 and 26. Qualitative interviews are conducted at 12 weeks (end of treatment) to ascertain their perceptions of treatment.

#### Primary outcome

##### Pain

Patient-rated low back pain at 12, 26, and 52 weeks, measured by the 11-box numerical rating scale, is the primary outcome measure. LBP at 4 and 8 weeks is a secondary outcome. Pain severity is regarded as one of the most important clinical outcomes by adults with spinal pain [33,34] and is recommended as a core outcome by an international group of back pain researchers in addition to the PedIMMPACT group [32,35]. The 11-box numerical rating scale for pain has been shown to perform similarly to the visual analogue scale (VAS) in both pediatric and adult populations [36,37].

#### Secondary outcomes

##### Disability

Patient-rated disability is assessed using the 18-item Roland-Morris Disability Questionnaire. The 18-item Roland-Morris Disability Questionnaire has been shown to be reliable, valid, and as responsive as the original 24-item Roland-Morris Disability Questionnaire [38,39].

##### Quality of life

Participants rate their quality of life using the 23-item PedsQL instrument, which has been developed to measure physical, emotional, social, and school functioning domains in children 8 to 18 years old. The PedsQL is a reliable, valid, and responsive measure of quality of life [40-43].

##### Improvement

Patient-rated improvement is determined by asking participants to compare their LBP condition to what it was before study treatment on a 9-point scale ranging from no symptoms (100% improvement) to as bad as it could be (100% worse). The caregiver’s impression of improvement will also be measured using the same 9-point scale. Improvement is an important outcome that has shown to be responsive [44].

##### Satisfaction

The participant’s overall satisfaction with care is assessed with a 7-point scale ranging from completely satisfied (couldn’t be better) to completely dissatisfied (couldn’t be worse). The caregiver’s satisfaction with care is also evaluated using the same scale. Research examining global satisfaction with treatment in pediatric pain trials is lacking and has been identified as an area of great need by the PedIMMPACT group [32].

##### Adverse events

The complete reporting of adverse events in clinical trials has received little attention [45]. To address this, we collect adverse event information at several levels. First, participants are queried about expected adverse events in the self-report questionnaires by choosing from a list generated from previous studies [46,47]. Participants are asked to rate the bothersomeness of each adverse event on a 0 to10 scale (0 = not at all bothersome, 10 = extremely bothersome). In addition, they are asked about new or ongoing adverse events at each treatment visit; these are recorded on standardized treatment notes. Finally, all patients are instructed to a report serious adverse event at any time to study staff (see Data and Safety Monitoring below).

##### Expectation

Participants rate their perception of how helpful they believe each treatment will be on a 1 to 5 scale (1 = Much better, 5 = Much worse) prior to randomization. After randomization, participants are asked to estimate the degree of improvement they expect to have after 12 weeks of care using the 9-point improvement scale described above.

##### Health care utilization and compliance

Patients report the number of visits and care received from non-study health care providers. They also report the number of days they took medication for LBP and performed the study exercises in the past week.

#### Qualitative outcomes

##### Qualitative interviews

One-on-one interviews are conducted at 12 weeks. A schedule of questions is used to direct the interviews and keep the interviewers on a path consistent with the purpose of the study [48]. The questions begin broadly, asking how patients felt about the treatment they received, whether it met their expectations, and what they liked and disliked. These questions are followed by probe questions to elicit underlying reasons.

#### Objective biomechanical outcomes

##### Continuous lumbar motion

Lumbar spinal motion is assessed using the Zebris CMS-HS Spine Motion Analyzer (Zebris Inc., Isny im Allgau, Germany), a reliable and accurate measurement system [49,50]. Lumbar flexion-extension, rotation, and side-bending are examined using a modified protocol described by Vogt et al [51].

##### Torso muscle strength & endurance

Endurance of the lower back musculature (trunk flexors, lateral flexors, and extensors) is examined using the protocol described by McGill [52]. The tests have been shown to be a valid and reliable measure of torso muscle endurance [53,54]. Maximum isometric strength of trunk flexors and extensors is measured using a modified standing method described by Jørgensen and Nicolaisen [55,56].

##### Activity level

Participants’ physical activity level is assessed by having them wear a GT3X accelerometer (Actigraph, Inc. Pensacola, FL) for 7 consecutive days prior to randomization, then again prior to the week 12 and 26 evaluations. The GT3X accelerometer has been found to be a reliable and valid measure of physical activity level [57].

### Analysis plan

For the primary analysis, patient-rated low back pain will be analyzed at 12, 26, and 52 weeks with linear mixed model regression to estimate mean differences between treatment groups at each time point adjusting for baseline covariates (i.e., rank-order minimization variables) and accounting for correlation across measurements within person. Short- and long-term longitudinal effects between groups will also be assessed using mixed model analyses including LBP intensity outcomes from weeks 4 to 12 and weeks 4 to 52, respectively. Due to the longitudinal study design, the primary analysis plan was modified from a one-way analysis of covariance to a linear mixed model approach to allow increased flexibility when specifying a variance-covariance structure and accounting for missing data [58-60]. The analysis plan was modified prior to the completion of data collection and the start of data analysis.

The variance-covariance structure that best fits the data will be used to account for correlation among the repeated measurements within participants [61,62]. Normality assumptions will be evaluated through normal probability plots and transformations used, if necessary. We will test for a site-by-group interaction, but given our standardization plan, we do not expect it to be present. Adjusted mean differences and 95% confidence intervals between groups for short- and long-term effects based on the final models will be presented in tables and line graphs. The need for inclusion of additional covariates to balance baseline group differences will be evaluated in sensitivity analysis. Intention-to-treat analysis will be used; all patients with at least one follow-up measure will be included in the analysis as the methods do not require data at every time point. In the event that missing data is present, the pattern of missingness will be assessed and appropriate imputation and sensitivity analyses will be performed. Responder analyses (i.e., 50, 75, and 100% pain reduction) will be conducted to facilitate the interpretation of the results [63-65].

### Power analysis and sample size

The basis for our sample size calculation is the one-way analysis of covariance (ANCOVA), comparing the primary outcome (reported pain level at the 12, 26, and 52 week time points) between groups and adjusting for baseline pain level. We estimated the standard deviation for change in pain using data from the trial by Jones et al [18] (SD = 1.4). A conservative R^2^ estimate of 0.2 was used for the sample size calculation. With a power of 0.92 and a 2-group design tested at an alpha level of 0.01, 80 subjects per group are required to detect a pain reduction of 0.8 points (Power and Precision™ 2.0). We will allow for a dropout rate/loss to follow-up of up to 15%. Therefore, 92 patients are required per group, for a total of 184 subjects.

### Data and safety monitoring

The Office of Data Management at Northwestern Health Sciences University serves as the data coordinating center responsible for creation of data collection forms, coordination of data transfer, and data management. A Data Safety and Monitoring Committee consisting of study and non-study clinicians and scientists meet annually to review the study. Due to the low risk profiles of the study interventions no interim analyses are planned. Adverse events are classified using the National Institutes of Health Office of Human Subjects Research definitions [66].

## Discussion

LBP is a significant health problem not only for adults, but also for children and adolescents. Given the large social and economic costs associated with the condition, identifying effective, conservative management strategies is critical. While SMT and exercise have been shown to be effective in adult populations, high quality research is needed to investigate their effectiveness in adolescent LBP patients.

Only a limited number of interventions for adolescent LBP have been evaluated in clinical trials; consequently, there is no established ‘gold-standard’ treatment. We chose exercise as a core intervention because of the evidence of effectiveness for adults with LBP [15,16], in addition to the preliminary evidence in adolescent populations [67]. Further, we wanted to encourage active behaviors for the management of pain in an adolescent population. We hypothesized that adding SMT to a promising treatment like exercise will produce greater improvements in LBP than what will be achieved by exercise alone.

Given there have been few studies addressing LBP in adolescents (and there have been no randomized clinical trials of SMT), we designed this trial with sufficient methodological rigor to maintain high internal validity; however, where possible, we also included pragmatic design features to enhance generalizability [68]. Explanatory design aspects of the trial aimed at ensuring high validity and low risk of bias include: the use of multiple baseline assessments to ensure participant compliance; eligibility criteria to exclude individuals unlikely to demonstrate treatment effects (e.g., pain intensity ≥ 3/10); restricted utilization of co-interventions during the intervention phase; structured protocols for the application of the intervention (number of visits, exercise, or SMT techniques used); application of the intervention at university-associated research clinics by clinicians with investigator supervision; and monitoring of participant and provider compliance including strategies to maximize adherence to study protocols. Pragmatic features of the study include: multiple recruitment sources from the general population at multiple sites; flexibility in the application of the intervention (e.g., exercise progressions, type of SMT used) based on individual patient needs and abilities; the use of an active treatment comparison group; the collection of both short- and long-term outcomes; and the plan to use intention-to-treat statistical analysis.

Upon completion, this study will provide high quality scientific evidence regarding SMT and exercise for LBP in adolescents with the potential to alter clinical practice and reduce both the current and future burden of LBP.

## Competing interests

The authors declare that they have no competing interests.

## Authors’ contributions

GB and MH are the co-principal investigators and have been primarily responsible for study conception, design, analysis plan, funding acquisition and implementation. CS, DP, DV, and RE contributed to the conceptualization and design of the study, funding acquisition, and implementation. BL participated in the implementation and management of the trial, drafted the Background, Methods, and Discussion sections of the manuscript, and coordinated manuscript preparation and revision. All authors provided critical evaluation and revision of the manuscript and have given final approval of the manuscript accepting responsibility for all aspects.
